# Physical Exercise Enhances Melatonin Effect in D-Galactose/Aluminum Chloride-Induced Alzheimer’s Disease of Ovariectomized Rats: Irisin Induction Associated with Upregulation of PPAR-γ/IGF-1/BDNF and Decreasing TNF-α/p38-MAPK/NLRP3/GFAP Pathway

**DOI:** 10.3390/ph19050770

**Published:** 2026-05-14

**Authors:** Ghada A. Badawi, Rawan S. Shaaban, Jawza A. Almutairi, Thanaa A. El-Masry, Hala F. Zaki, Sherehan M. Ibrahim

**Affiliations:** 1 Department of Pharmacology and Toxicology, Faculty of Pharmacy, Sinai University-Arish Branch, Arish 45511, Egypt; ghada.ali@su.edu.eg (G.A.B.); rawan.saied@su.edu.eg (R.S.S.); thanaa.ahmed@su.edu.eg (T.A.E.-M.); 2 Department of Pharmaceutical Sciences, College of Pharmacy, Princess Nourah Bint Abdulrahman University, P.O. Box 84428, Riyadh 11671, Saudi Arabia; 3 Department of Pharmacology and Toxicology, Faculty of Pharmacy, Tanta University, Tanta 31527, Egypt; 4 Department of Pharmacology and Toxicology, Faculty of Pharmacy, Cairo University, Cairo 11562, Egypt; hala.fahmy@pharma.cu.edu.eg (H.F.Z.); sherehan.mohamed@pharma.cu.edu.eg (S.M.I.); 5 Department of Pharmacology and Toxicology, Faculty of Pharmacy, Modern University for Technology and Information, Cairo 11571, Egypt

**Keywords:** irisin, TNF-α, p38 MAPK, PPAR-γ, NLRP3, GFAP

## Abstract

**Background**: Postmenopausal women are at high risk of Alzheimer’s disease (AD) incidence and progression. Irisin, an exercise-induced myokine, has neuroprotective and antiaging effects against AD, especially in menopausal women suffering from insulin resistance (IR). For the first time, the novel role of irisin induced by melatonin (MTN) or/and physical exercise (PHE) was investigated in the current ovariectomized (OVX)/AD rat model by modulating brain neuroinflammation and IR-related markers. **Methods**: Fifty female Wistar rats were divided into five groups, with one representing a sham group. AD was induced in the other four bilateral OVX rat groups by daily intraperitoneal injection of D-galactose/AlCl_3_ (60 and 10 mg/kg, respectively) for 42 days. Group III–V: Animals were exposed to MTN (10 mg/kg/day; i.p.), PHE, and a combination of these, respectively, in the final 14 days of the experiment. **Results**: The OVX/AD rats showed significant deterioration in learning, memory, neurochemical, and histopathological examinations, while the MTN or/and PHE treatments significantly increased serum and brain irisin, improving memory in a Y-maze assessment. Thus, hippocampal histopathological alterations and IR-related markers decreased. In addition, suppressed hippocampal amyloid-beta protein expression and neuroinflammatory content of tumor necrosis factor-alpha (TNF-α), p38 mitogen-activated protein kinase (p38 MAPK), and NOD-like receptor protein-3 (NLRP3) were associated with an increase in peroxisome proliferator-activated receptor-gamma (PPAR-γ) protein expression and insulin-like growth factor-1 content in hippocampal tissues, collectively suppressing glial fibrillary acidic protein (GFAP) content, leading to an increase in brain-derived neurotrophic factor expression. **Conclusions**: Irisin induction may serve as a novel avenue in AD/menopause treatment and prevention via modulating the TNF-α/p38 MAPK/PPAR-γ/NLRP3/GFAP pathway.

## 1. Introduction

Alzheimer’s disease (AD) is a neurodegenerative disease that progressively damages learning and memory, and is characterized by intraneuronal buildup of hyperphosphorylated tau protein and extracellular deposition of amyloid beta (Aβ) [1].

Decline in memory and cognition is a known complication in postmenopausal women [2]. According to data from the World Health Organization (WHO), about fifty-five million people worldwide suffered from dementia in 2021 (5.4% of men vs. 8.1% of women over 65 years) [3,4].

There is growing evidence that estradiol deprivation after menopause or ovariectomy is a risk factor closely related to the pathological development of AD, as there is an appreciable increase in brain acetylcholine esterase activity and a concomitant decrease in acetylcholine level in ovariectomized (OVX) rats [5,6]. Ovariectomy induced a shift in brain energy metabolism characterized by reduced glucose uptake and metabolism in the hippocampus, contributing to impaired neuronal bioenergetics and increased Aβ oligomer levels [7].

Several studies have shown that neuroinflammation and insulin resistance (IR) modulate the pathogenesis of both AD and menopause [8]. Importantly, tumor necrosis factor-alpha (TNF-α) functions as a pro-inflammatory cytokine that plays a pivotal role in AD pathogenesis and progression. Elevated TNF-α levels are consistently observed in AD patients and are intricately involved in the formation of Aβ plaques and other key pathological features of the disease [9].

Importantly, the inflammatory cascade activated by TNF-α leads to significant stimulation of the inflammatory pathway of p38 mitogen-activated protein kinase (p38 MAPK), which is associated with the augmented expression of NOD-like receptor protein 3 (NLRP3) and other downstream inflammatory mediators. The p38 MAPK pathway acts as a critical signaling mechanism in AD, as phosphorylated p38 MAPK is markedly increased in AD brains and localizes within Aβ plaques. This triggers a dramatic increase in glial fibrillary acidic protein (GFAP), a condition recognized as astrocytopathy [10,11,12].

Notably, IR is strongly related to memory dysfunction and AD, highlighting insulin’s crucial function in memory processing and broader brain activity [13,14]. Consequently, decreasing IR may provide therapeutic benefits for mitigating AD progression. Peroxisome proliferator-activated receptor gamma (PPAR-γ) functions as a multifaceted transcription factor, with anti-inflammatory activity in addition to its essential regulatory roles in numerous vital biological processes [15,16].

Irisin, an exercise-generated myokine, was discovered in 2012; serving as a messenger between adipocytes and skeletal muscles, it represents a potential bearer of the benefits of physical exercise (PHE) on many target tissues other than muscle [17]. It is derived from fibronectin (type III)-domain-containing 5 (FNDC5), a muscle transmembrane precursor protein that is regulated by PPAR-γ co-activator-1α. Interestingly, FNDC5 is strongly distributed in different areas of the brain that are responsible for memory and spatial learning [18]. Furthermore, several studies have shown irisin’s neuroprotective activity via its anti-inflammatory, anti-apoptotic, antioxidant, and neurotrophic efficacy via modulating brain metabolic activity and improving glucose homeostasis [19,20,21].

It is proposed that decreasing female sex hormones in menopause or OVX animals decreases irisin levels and suppresses skeletal muscle production of PPAR-γ coactivator-1α and FNDC5, supporting high brain susceptibility to AD incidence and progression [22]. Therefore, irisin induction may suppress brain neuroinflammation, IR and its related markers that modulate AD pathological cascades.

Irisin levels are generally reduced in models of neurodegenerative disorders like AD and Parkinson’s disease, and exogenous irisin administration has been demonstrated to dramatically lower oxidative stress levels in the brain and improve mitochondria function [23,24].

Melatonin (MTN) is a hormone of the pineal gland; it modulates different body and cellular processes, in addition to circadian rhythm regulation. Importantly, MTN represents a potential sign of AD, as it gradually declines in AD modeling [25,26]. Many studies have shown that MTN demonstrates neurogenesis, neuroprotection, and neurotherapeutic efficacy via several mechanistic pathways, i.e., anti-inflammatory, anti-apoptotic, and antioxidant activity [27,28,29]. Numerous studies have shown that MTN enhances circulating irisin, through which it can modulate several vital processes [30,31,32]. Overnight MTN secretion promotes diurnal insulin sensitivity peripherally [33] and ameliorates brain IR in brain aging and cognition [34].

Recent studies have elaborated that individual treatment of either MTN or PHE increases irisin, which has neuroprotective effects in AD [35,36,37,38]. However, the current work investigated for the first time the effect of merging pharmacological (MTN) and non-pharmacological (PHE) treatments in OVX/AD rat modeling by the induction of endogenous irisin, associated with upregulation of PPAR-γ/insulin-like growth factor 1 (IGF-1)/brain-derived neurotrophic factor (BDNF) and reduction in the TNF-α/p38 MAPK/NLRP3/GFAP pathway.

## 2. Results

Although there were significant differences among the different groups, the current work focused on the significant difference between the sham vs. OVX/AD group; OVX/AD vs. groups treated by PHE, MTN, or combination treatments; and the combined treatment vs. individual treatment groups.

### 2.1. Effects of MTN and PHE Either Alone or in Combination on Rats’ Cognition and Behaviors in Y-Maze Test in OVX/AD Rats

**Figure 1:**
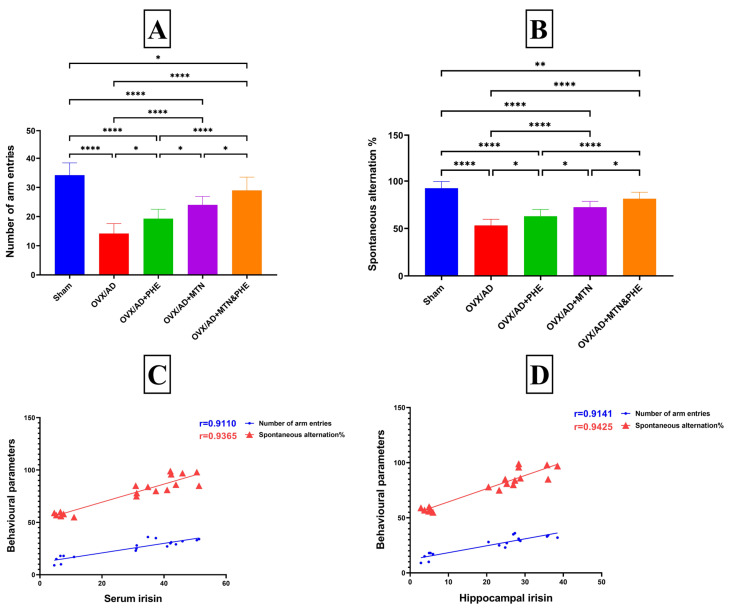
Effects of MTN (10 mg/kg/day; i.p) and PHE alone or in combination on rats’ behaviors and cognition in Y-maze test in OVX/AD rats. (**A**) number of arm entries in the Y-maze test, (**B**) spontaneous alternation percentage in the Y-maze test, (**C**) correlation between serum concentration of irisin and both number of arm entries and spontaneous alternation percentage, and (**D**) correlation between hippocampal content of irisin and both number of arm entries and spontaneous alternation percentage. Data are presented as mean ± SD (*n* = 10). Statistical analysis was conducted by GraphPad Prism, one-way ANOVA followed by Tukey post hoc test for multiple comparisons; (*) *p* ≤ 0.05, (**) *p* ≤ 0.01, (****) *p* ≤ 0.0001. Correlation analysis was performed using Pearson’s correlation coefficient. AD: Alzheimer’s disease; ANOVA: analysis of variance; MTN: melatonin; OVX: ovariectomized; PHE: physical exercise.

The OVX rats that received D-glc/AlCl_3_ showed significant deterioration in spatial working memory and cognition, as they exhibited a significant reduction in the number of arm entries by about 58% and a decrease in SA percentage by 42% when compared to the sham group. Interestingly the MTN or/and PHE animals displayed a significant increase in the number of arm entries by about 69%, 36%, and 104%, respectively, and an increase in SA percentage by 36%, 18%, and 53%, respectively, when compared to the OVX/AD rats, with a greater effect in the group receiving combined treatments (Figure 1A,B).

Notably, significant positive correlations between irisin serum concentration and behavioral parameters were detected, as shown by the number of arm entries (r = 0.8181, *p* ≤ 0.0001_FDR_, η^2^ = 0.6693) and SA percentage (r = 0.8164, *p* ≤ 0.0001_FDR_, η^2^ = 0.6665). Similarly, the same correlations were shown between the hippocampal content of irisin and behavioral parameters of the number of arm entries (r = 0.8100, *p* ≤ 0.0001_FDR_, η^2^ = 0.6561) and SA percentage (r = 0.8091, *p* ≤ 0.0001_FDR_, η^2^ = 0.6574) (Figure 1C,D).

### 2.2. Effects of MTN and PHE Either Alone or in Combination on Serum Irisin Concentration and Hippocampal Irisin Content in OVX/AD Rats

**Figure 2:**
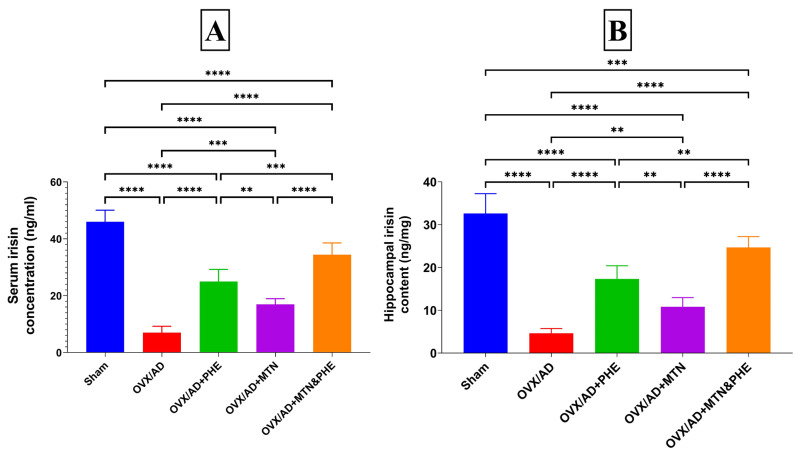
Effects of MTN (10 mg/kg/day; i.p.) and PHE alone or in combination on (**A**) serum irisin concentration and (**B**) hippocampal irisin content in OVX/AD rats. Data are presented as mean ± SD (*n* = 6). Statistical analysis was conducted by GraphPad Prism, one-way ANOVA followed by Tukey–Kramer as a post hoc test for multiple comparisons; (**) *p* ≤ 0.01, (***) *p* ≤ 0.001, (****) *p* ≤ 0.0001. AD: Alzheimer’s disease; ANOVA: analysis of variance; MTN: melatonin; OVX: ovariectomized; PHE: physical exercise.

The OVX/AD group displayed a significant decrease in serum and hippocampal content of irisin by 15% and 14%, correspondingly, as compared to the sham group. The mean difference between the sham and OVX/AD groups for serum irisin was (39.01, [95% CI of difference 33.09 to 44.94, *p* < 0.0001_FDR_]) and hippocampal irisin (28.01, *p* < 0.0001_FDR_). Conversely, MTN or/and PHE treatments increased the serum concentration of irisin by about 1.5-, 2.5-, and 4-fold, correspondingly. The serum irisin mean difference (η^2^ = 0.9472) between the OVX/AD and OVX/AD+MTN groups was (−9.923, *p* < 0.0001_FDR_), the OVX/AD and OVX/AD+PHE groups was (−17.96, *p* < 0.0001_FDR_), and the OVX/AD and OVX/AD+MTN&PHE groups was (−27.40, *p* < 0.0001_FDR_) (Figure 2A). Similarly, they increased hippocampal content of irisin by 1.5-, 2.5-, and 4.5-fold, correspondingly, as compared to the OVX/AD group, with superior results for combination treatments of MTN and PHE. The hippocampal irisin mean difference (η^2^ = 0.9314) between the OVX/AD and OVX/AD+MTN groups was (−6.198, *p* = 0.0013_FDR_), the OVX/AD and OVX/AD+PHE groups was (−12.71, *p* < 0.0001_FDR_), and the OVX/AD and OVX/AD+MTN&PHE groups was (−20.06, *p* < 0.0001_FDR_) (Figure 2B).

### 2.3. Effects of MTN and PHE, Either Alone or in Combination, on Number of Positive Neurons of Hippocampal BDNF and Percent of Positive Hippocampal Aβ Deposits in OVX/AD Rats

**Figure 3:**
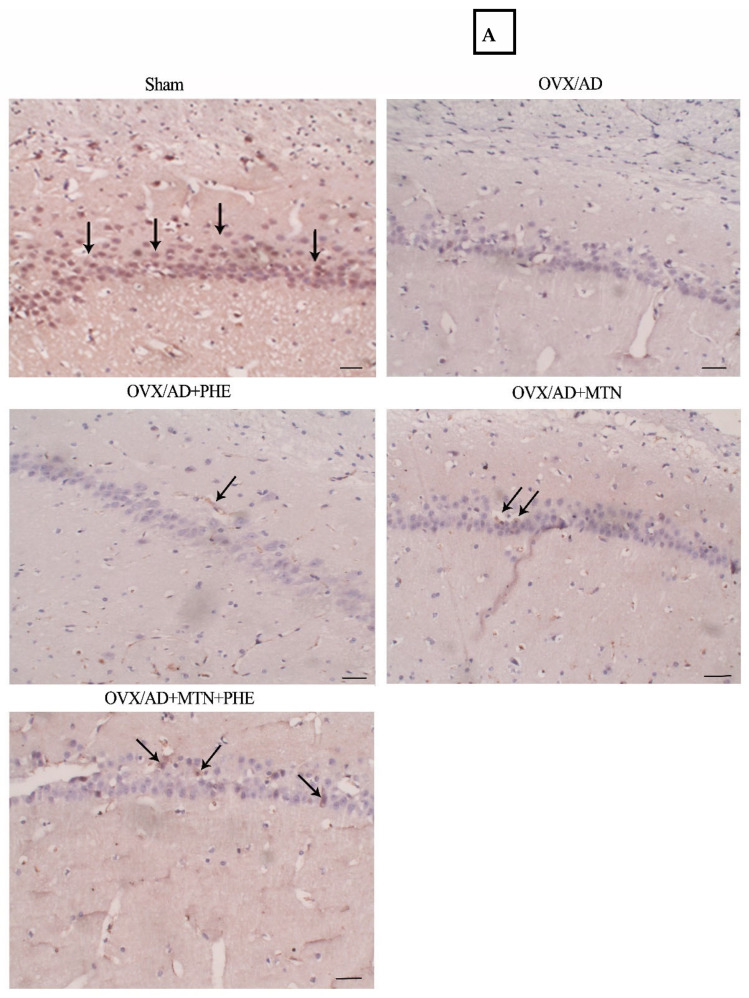

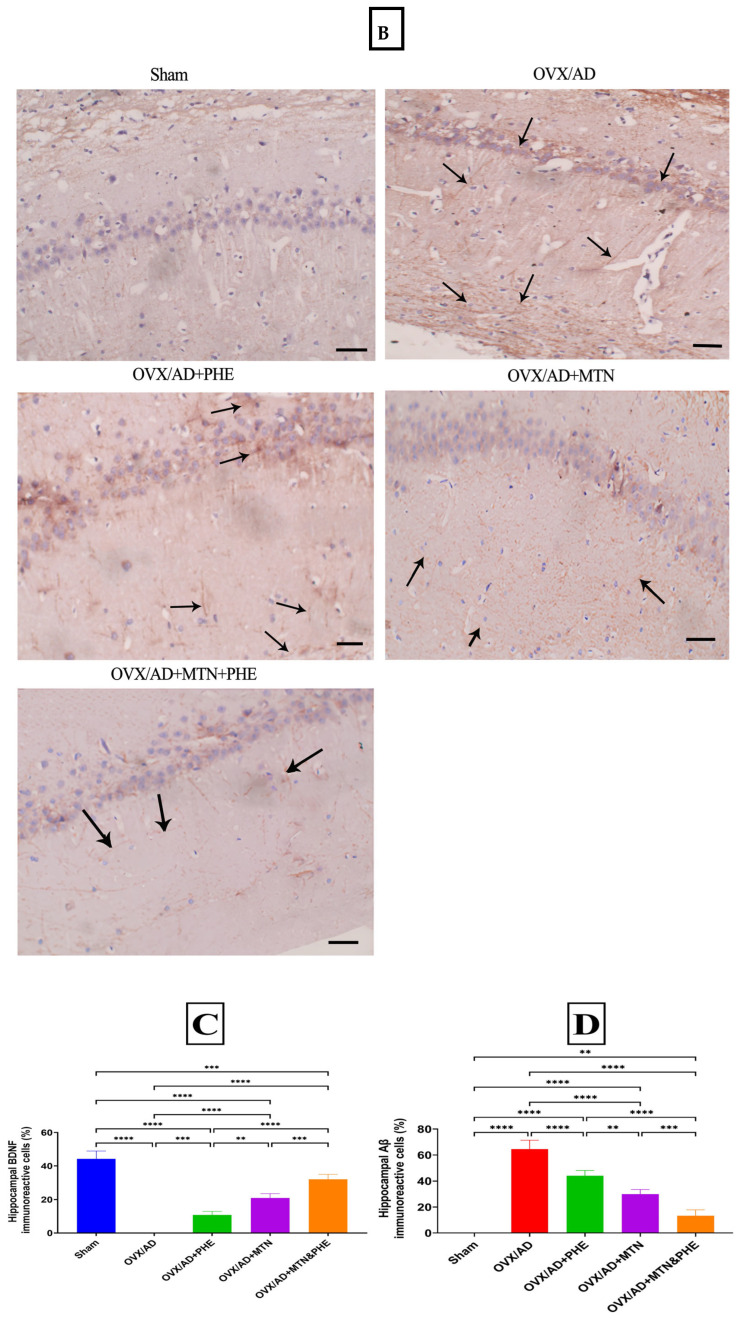
Effects of MTN (10 mg/kg/day; i.p.) and PHE alone or in combination on BDNF and Aβ protein expression cells in OVX/AD rats. (**A**) Photomicrographs of the hippocampus subiculum sections immunostained with BDNF, scale bar = 50 μm (200×) (black arrows represent the number of positive neurons of hippocampal BDNF). (**B**) Photomicrographs of the hippocampus subiculum sections immunostained with Aβ, scale bar = 50 μm (200×) (black arrows represent the percent of positive hippocampal Aβ deposits). (**C**) Number of positive neurons of hippocampal BDNF, and (**D**) percent of positive hippocampal Aβ deposits. Data are presented as mean ± SD (*n* = 4). Statistical analysis was conducted by GraphPad Prism, one-way ANOVA followed by Tukey–Kramer as a post hoc test for multiple comparisons; (**) *p* ≤ 0.01, (***) *p* ≤ 0.001, (****) *p* ≤ 0.0001. AD: Alzheimer’s disease; MTN: melatonin; OVX: ovariectomized; PHE: physical exercise.

The OVX/AD rats showed a marked reduction in the number of positive neurons of hippocampal BDNF in the rats’ hippocampal subiculum when compared with the sham group. The mean difference between the sham and OVX/AD groups for number of positive neurons of hippocampal BDNF was (44.25, *p* < 0.0001_FDR_). The animals that received MTN or/and PHE treatments exhibited a significant increase in the number of positive neurons of hippocampal BDNF by two-fold, one-fold, as well as three-fold, respectively, in their hippocampal subiculum as compared to the OVX/AD group. Additionally, the combined treatment showed a better effect regarding increasing the number of positive neurons of hippocampal BDNF when compared to individual treatments, as detected by the immune staining technique. The number of positive neurons of hippocampal BDNF mean difference (η^2^ = 0.9746) between the OVX/AD and OVX/AD+MTN groups was (−21.00, *p* < 0.0001_FDR_), the OVX/AD and OVX/AD+PHE groups was (−10.75, *p* = 0.0001_FDR_), and the OVX/AD and OVX/AD+MTN&PHE groups was (−32.00, *p* < 0.0001_FDR_) (Figure 3A,C).

In the same context, the % of positive hippocampal Aβ deposits was upregulated in the OVX/AD animals by 6.5-fold as compared to the sham group. The mean difference between the sham and OVX/AD groups for % of positive hippocampal Aβ deposits was (−64.50, *p* < 0.0001_FDR_). Moreover, the administration of MTN and PHE, either alone or in combination, exhibited a marked decrease of the (%) of positive hippocampal Aβ deposits by 53%, 32%, and 79% respectively, as compared to the OVX/AD rats. Furthermore, the combined treatment presented an enhanced effect regarding decreasing the percent of positive hippocampal Aβ deposits when compared to individual treatments, as detected by the immunostaining technique. The (%) of positive hippocampal Aβ deposits mean difference (η^2^ = 0.9724) between the OVX/AD and OVX/AD+MTN groups was (34.50, *p* < 0.0001_FDR_), the OVX/AD and OVX/AD+PHE groups was (20.50, *p* < 0.0001_FDR_), and the OVX/AD and OVX/AD+MTN&PHE groups was (51.25, *p* < 0.0001_FDR_) (Figure 3B,D).

### 2.4. Effects of MTN and PHE, Either Alone or in Combination, on Number of Positive Neurons of Hippocampal PPAR-γ and IGF-1 Content in OVX/AD Rats

**Figure 4:**
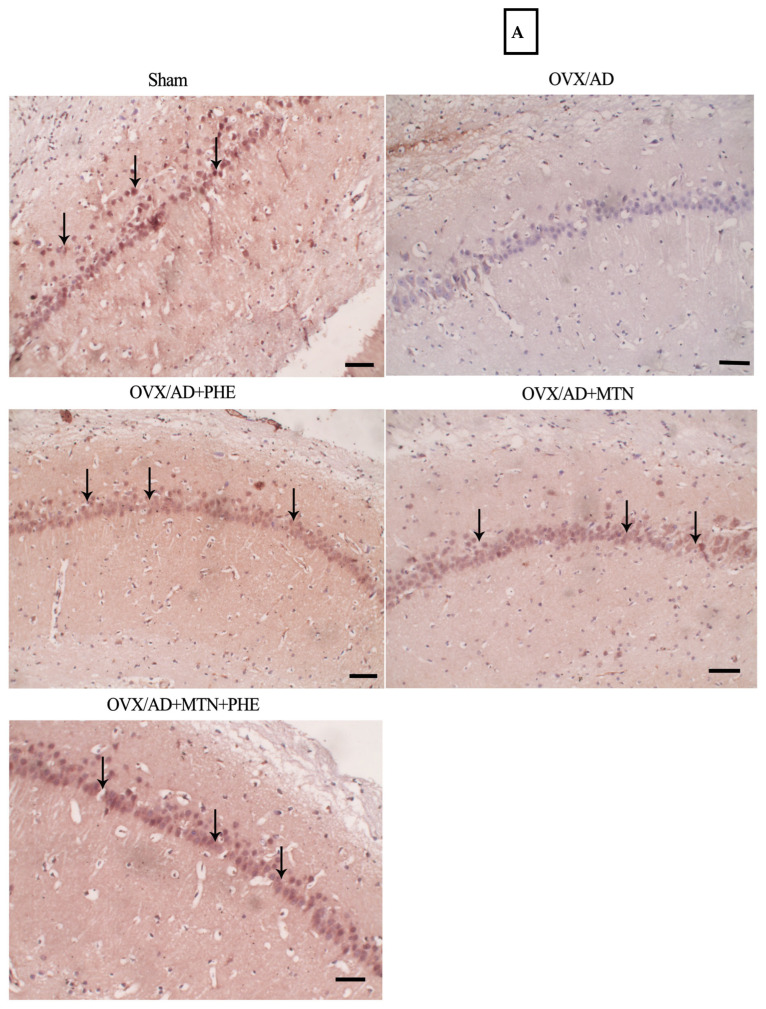

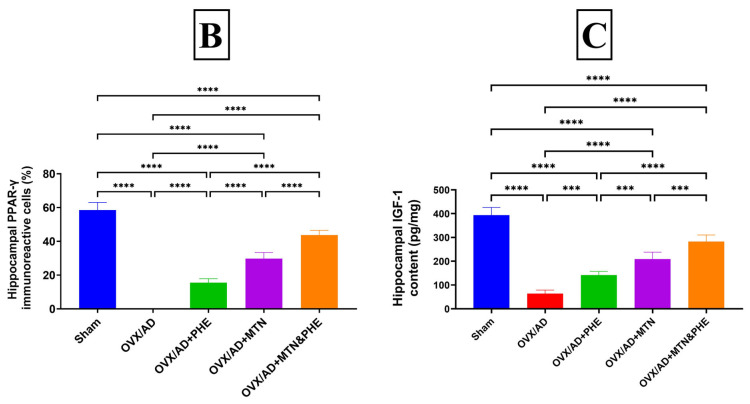
Effects of MTN (10 mg/kg/day; i.p.) and PHE, alone or in combination, on PPAR-γ protein expression and IGF-1 content in OVX/AD rats. (**A**) Photomicrographs of the hippocampus subiculum sections immunostained with PPAR-γ, scale bar = 50 μm (200×) (black arrows represent the number of positive neurons of hippocampal PPAR-γ). (**B**) Number of positive neurons of hippocampal PPAR-γ, and (**C**) hippocampal content of IGF-1. Data are presented as mean ± SD (*n* = 4 and 6 for PPAR-γ and IGF-1, respectively). Statistical analysis was conducted by GraphPad Prism, one-way ANOVA followed by Tukey–Kramer as a post hoc test for multiple comparison; (***) *p* ≤ 0.001, (****) *p* ≤ 0.0001. Correlation analysis was performed using Pearson’s correlation coefficient. AD: Alzheimer’s disease; ANOVA: analysis of variance; IGF-1: insulin growth factor; MTN: melatonin; OVX: ovariectomized; PPAR-γ: peroxisome proliferator-activated receptor gamma; PHE: physical exercise.

The number of positive neurons of hippocampal PPAR-γ in the OVX/AD rats’ hippocampus subiculum were significantly decreased when compared to the sham group. The mean difference between the sham and OVX/AD groups for the number of positive neurons of hippocampal PPAR-γ was (58.50, *p* < 0.0001_FDR_). The animals administered either MTN or PHE or combined treatments showed significant increases in the number of positive neurons of hippocampal PPAR-γ by three-fold, 1.5-fold, and 4.5-fold, respectively, as compared to the OVX/AD rats. Furthermore, the combined treatment showed an enhanced effect regarding increasing the number of positive neurons of hippocampal PPAR-γ when compared to individual treatments. The number of positive neurons of hippocampal PPAR-γ mean difference (η^2^ = 0.9837) between the OVX/AD and OVX/AD+MTN groups was (−29.75, *p* < 0.0001_FDR_), the OVX/AD and OVX/AD+PHE groups was (−15.50, *p* < 0.0001_FDR_), and the OVX/AD and OVX/AD+MTN&PHE groups was (−43.75, *p* < 0.0001_FDR_) (Figure 4A,B).

In the same context, the OVX/AD rats showed a marked downregulation in brain content of IGF-1 to 16% comparable to the sham group. The mean difference between the sham and OVX/AD groups for hippocampal IGF-1 was (329.8, *p* < 0.0001_FDR_), while the rats administered MTN or/and PHE treatments exhibited a significant upregulation in brain content of IGF-1 to 329%, 223%, and 446%, respectively, when compared to the OVX/AD animals. In addition, the combined treatment showed an enhanced effect regarding increasing IGF-1 expression when compared to the individual treatments. The hippocampal IGF-1 mean difference (η^2^ = 0.9605) between the OVX/AD and OVX/AD+MTN groups was (−145.5, *p* = 0.0013_FDR_), the OVX/AD and OVX/AD+PHE groups was (−77.74, *p* < 0.0001_FDR_), and the OVX/AD and OVX/AD+MTN&PHE groups was (−219.0, *p* < 0.0001_FDR_) (Figure 4C).

### 2.5. Effects of MTN and PHE, Either Alone or in Combination, on Pro-Inflammatory Biomarkers in OVX/AD Rats

**Figure 5:**
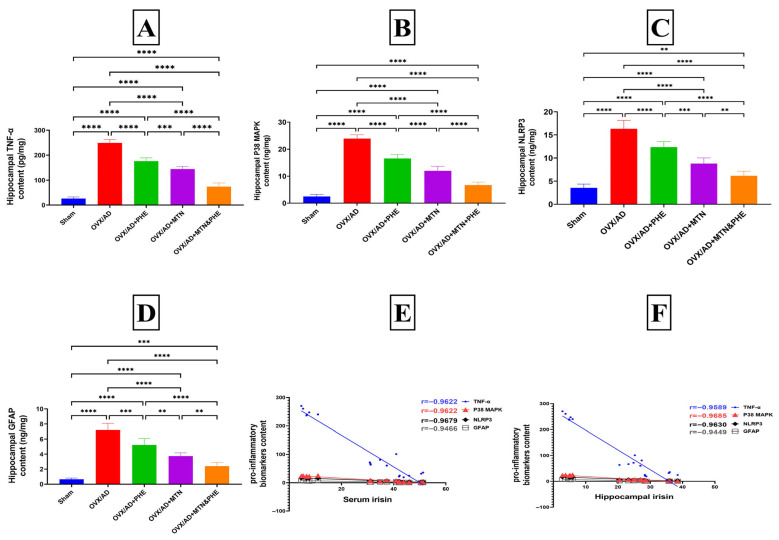
Effects of MTN (10 mg/kg/day; i.p) and PHE, alone or in combination, on pro-inflammatory biomarkers of hippocampi of OVX/AD rats. (**A**) TNF-α content, (**B**) P38 MAPK content, (**C**) NLRP3 content, (**D**) GFAP content, (**E**) correlation between serum concentration of irisin and hippocampal content of TNF-α, P38 MAPK, NLRP3, and GFAP inflammatory biomarker content, and (**F**) correlation between hippocampal content of irisin and hippocampal content of TNF-α, P38 MAPK, NLRP3, and GFAP inflammatory biomarker content. Data are presented as mean ± SD (*n* = 6). Statistical analysis was conducted by GraphPad Prism, one-way ANOVA followed by Tukey–Kramer as a post hoc test for multiple comparisons; (**) *p* ≤ 0.01, (***) *p* ≤ 0.001, (****) *p* ≤ 0.0001. Correlation analysis was performed using Pearson’s correlation coefficient. AD: Alzheimer’s disease; ANOVA: analysis of variance; GFAP: glial fibrillary acidic protein; MTN: melatonin; NLRP3: NOD-like receptor protein 3; OVX: ovariectomized; PHE: physical exercise; p38 MAPK: p38 mitogen-activated protein kinase; TNF-α: tumor necrosis factor alpha.

The OVX/AD rats showed a marked increase in brain hippocampal content of pro-inflammatory biomarkers of TNF-α, p38 MAPK, NLRP3, and GFAP by about 8.5-fold, 8.5-fold, 3.5-fold, and 10-fold, correspondingly, as compared to the sham group. The mean difference between the sham and OVX/AD groups for TNF-α was (−222.8, *p* < 0.0001_FDR_), p38 MAPK (−21.38, *p* < 0.0001_FDR_), NLRP3 (−12.80, *p* < 0.0001_FDR_), and GFAP (−6.544, *p* < 0.0001_FDR_). Furthermore, the animals that received either MTN or PHE or combined treatments showed a significant decline of hippocampal content of TNF-α by about 42%, 29%, and 70%, respectively; the TNF-α mean difference (η^2^ = 0.9802) between the OVX/AD and OVX/AD+MTN groups was (104.9, *p* < 0.0001_FDR_), the OVX/AD and OVX/AD+PHE groups was (72.86, *p* < 0.0001_FDR_), and the OVX/AD and OVX/AD+MTN&PHE groups was (175.3, *p* < 0.0001_FDR_). There was a marked decline in hippocampal p38 MAPK content by 50%, 31%, and 72%, respectively; the p38 MAPK mean difference (η^2^ = 0.9747) between the OVX/AD and OVX/AD+MTN groups was (11.90, *p* < 0.0001_FDR_), the OVX/AD and OVX/AD+PHE groups was (7.328, *p* < 0.0001_FDR_), and the OVX/AD and OVX/AD+MTN&PHE groups was (17.20, *p* < 0.0001_FDR_). There was a notable decrease in brain content of the NLRP3 content by about 46%, 24%, and 62%, respectively; the NLRP3 mean difference (η^2^ = 0.9395) between the OVX/AD and OVX/AD+MTN groups was (7.511, *p* < 0.0001_FDR_), the OVX/AD and OVX/AD+PHE groups was (3.971, *p* < 0.0001_FDR_), and the OVX/AD and OVX/AD+MTN&PHE groups was (10.16, *p* < 0.0001_FDR_). And there was a noticeable reduction in hippocampal GFAP content by 47%, 27%, and 66%, respectively; the GFAP mean difference (η^2^ = 0.9392) between the OVX/AD and OVX/AD+MTN groups was (3.446, *p* < 0.0001_FDR_), the OVX/AD and OVX/AD+PHE groups was (1.969, *p* < 0.0001_FDR_), and the OVX/AD and OVX/AD+MTN&PHE groups was (4.775, *p* < 0.0001_FDR_), when compared to the OVX/AD rats, with the greatest effect of the combined treatments (Figure 5A,D).

It is noteworthy that significant negative correlations were detected between irisin serum level and pro-inflammatory biomarkers content, as follows: TNF-α (r = −0.8954, *p* ≤ 0.0001_FDR_, η^2^ = 0.8018), p38 MAPK (r = −0.8748, *p* ≤ 0.0001_FDR_, η^2^ = 0.7652), NLRP3 (r = −0.8534, *p* ≤ 0.0001_FDR_, η^2^ = 0.7283), and GFAP (r = −0.8460, *p* ≤ 0.0001_FDR_, η^2^ = 0.7157). Similarly, significant negative correlations were detected between the hippocampal content of irisin and pro-inflammatory biomarker content, as follows: TNF-α (r = −0.8859, *p* ≤ 0.0001_FDR_, η^2^ = 0.7848), P38 MAPK (r = −0.8678, *p* ≤ 0.0001_FDR_, η^2^ = 0.7531), NLRP3 (r = −0.8380, *p* ≤ 0.0001_FDR_, η^2^ = 0.7022), and GFAP (r = −0.8319, *p* ≤ 0.0001_FDR_, η^2^ = 0.6921) (Figure 5E,F).

### 2.6. Effects of MTN and PHE, Either Alone or in Combination, on Brain Histopathological Changes in OVX/AD Rats

**Figure 6:**
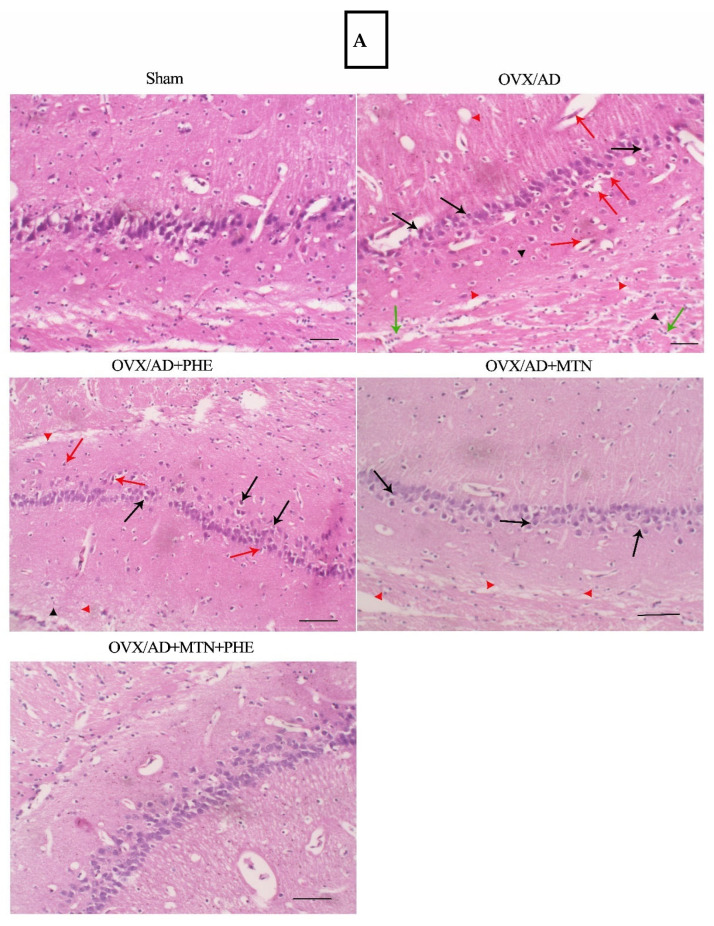

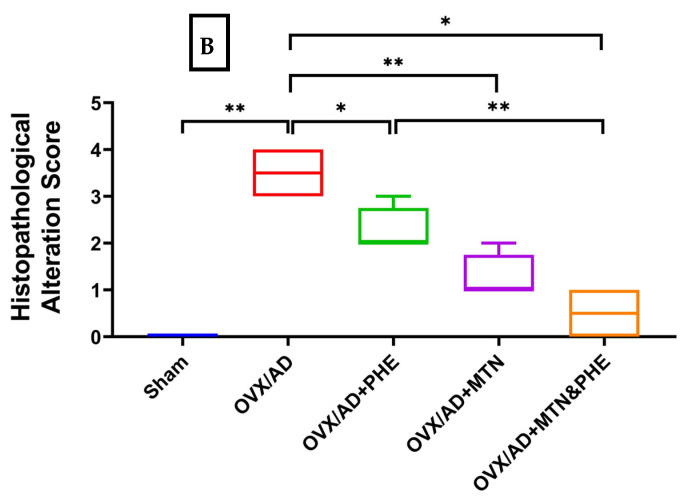
Effects of MTN (10 mg/kg/day; i.p.) and PHE, alone or in combination, on histopathological changes in hippocampal subiculum of OVX/AD rats. (**A**) Photomicrographs showing the histopathological alteration in hippocampal subiculum sections, scale bar = 50 μm (200×) [black arrow: indicates nuclear pyknosis and peri-neuronal edema; red arrows: red neurons; green arrows: aggregates of lymphocytic infiltrate; red arrowheads: tissue edema; black arrowheads: areas of gliosis], and (**B**) graph showing the histopathological alteration score; scoring of hippocampal histopathological alteration tissues was estimated in the following manner, (grade 0: absent; grade 1: minimal; grade 2: mild; grade 3: moderate; grade 4: marked), then the mean number of scores in each group was calculated separately and compared among different groups. Data are presented as median (Min to Max) ± SD (*n* = 4). Statistical analysis was conducted by GraphPad Prism, employing the Kruskal–Wallis test. Pairwise comparisons were carried out using the Mann–Whitney U test; (*) *p* ≤ 0.05, (**) *p* ≤ 0.01. AD: Alzheimer’s disease; MTN: melatonin; OVX: ovariectomized; PHE: physical exercise.

The histopathological examination of the hippocampal subiculum sections of sham animals stained by H&E indicated normal tissue architecture, while the OVX/AD rats showed extensive expression of pyknotic nuclei, gliosis, peri-neuronal edema, and chronic inflammatory cell infiltrates, which indicated a progressive degeneration of hippocampal tissues. The OVX/AD rats treated by MTN or PHE showed mild gliosis and decreased edema and inflammation. Remarkably, MTN co-treatment with PHE showed significant repair of hippocampal neurons as compared to the OVX/AD animals, as there was no evidence of edema, gliosis, or neuronal injury (Figure 6A). Furthermore, the OVX/AD rats treated by MTN or PHE, or combined treatments, decreased the histopathological alteration scores significantly by 63%, 37%, and 84%, respectively, as compared to the OVX/AD rats (Figure 6B).

## 3. Discussion

AD is a worldwide public health challenge, as the primary cause of the severe impairment of cognitive function known as dementia. Urgently, several investigations are needed to explore strategies that slow and prevent AD progression. Importantly, the main causes of AD are the accumulation of Aβ protein and neurofibrillary tangles within the brain [39,40].

Many studies have confirmed that animal models with D-glc and AlCl_3_ together showed clinical signs similar to AD features in humans, reflected by high expression levels of Aβ protein aggregates, the formation of senile plaques, and neurofibrillary tangles [41,42].

Interestingly, AlCl_3_ accumulates in diverse brain areas, i.e., the cerebellum, hippocampus, and cortex [43,44], which share in cognition and memory functions, via Aβ accumulation, lipid peroxidation impairment, and central neurotransmission [45,46]. It can activate a series of inflammatory pathways that act as promoters of neuroinflammation, IR, and related neurodegenerative processes [47,48].

Additionally, D-glc, a neurotoxicant and sugar in nature, is widely used in AD modeling as it can cause cognition and memory deficits. At high doses, it yields abnormal aldehyde metabolites that accumulate in the brain and deteriorate the antioxidant defense system [49]. In addition, it induces neuroinflammation, which augments the AD neurodegenerative process [50,51].

Interestingly, postmenopausal women experience a higher susceptibility to AD incidence and progression, attributed to the termination of ovarian estrogen production, hence accelerating brain neurodegeneration. Thus, estrogen depletion can lead to AD by triggering neuroinflammation, suppressing neurotrophic factors, and increasing the brain content of Aβ proteins [52]. Therefore, the OVX animals receiving D-glc/AlCl_3_ represent an effective model that mimics biochemical, pathophysiological, and behavioral features similar to those in postmenopausal women with AD [22,53].

The administration of D-glc/AlCl_3_ to the OVX rats in the current work showed an overall decrease in cognition and the biochemical and histopathological features of hippocampal biomarkers, as compared to the sham animals. However, the OVX/AD group showed high hippocampal Aβ protein expression, accompanied by a significant decrease in BDNF protein expression, which parallels the studies of [54,55], who illustrated that ovariectomy reduced hippocampal BDNF content and increased amyloidogenesis, leading to an exaggeration of amyloid plaque, which in turn restricted hippocampal elasticity and neurotrophic support of BDNF.

In addition, the OVX/AD animals exhibited a marked increase in brain pro-inflammatory biomarker content, i.e., TNF-α, NLRP3, and GFAP, that parallels that found in [55] and may be attributed to the significant increase in Aβ deposits, eliciting neuroinflammation through astrocyte recruitment, as well as microglia around senile plaques and inflammatory cytokine liberation. In many neuropathological conditions, TNF-α functions as a key mediator of brain neuroinflammation and various tissues [56]. Bauernfeind et al. (2016) showed that TNF-α activates NLRP3 and other inflammatory pathways, which in turn lead to a series of inflammatory cascades [10].

Furthermore, the OVX/AD animals exhibited a marked upregulation in brain p38 MAPK with a notable reduction in BDNF protein expression, thus initiating the neurodegenerative process; previous studies have reported that a significant increase in brain p38 MAPK content leads to a significant decline in BDNF in OVX0AD rats [57,58]. Moreover, the OVX/AD rats showed noticeable histopathological alterations, reflecting the extent of hippocampal tissue damage and neuronal loss, manifested as an increase in pyknotic neuronal cells, gliosis, and edema in hippocampal tissues, which paralleled the previous study of [42]. Together, the measured neurochemical and pathological parameters in the hippocampal tissue explain the deterioration of animal learning and memory that was observed in the Y-maze test, as the OVX/AD rats presented a significant reduction in both spatial working memory and number of arm entries, along with inhibition of SA percentage when compared to the sham rats, which paralleled the previous studies of [59,60].

Herein, the OVX/AD animals displayed a noteworthy reduction in their serum and brain irisin content, associated with a marked decrease in both PPAR-γ protein expression and IGF-1 content in the hippocampal tissue; this was in accordance with previous evidence [61] demonstrating that irisin levels were downregulated along with a distinguished reduction in the hippocampal levels of PGC-1α and BDNF in the OVX/AD animals.

Irisin is represented as an adipomyokine, induced by exercise or body hormones such as MTN. It has both myogenic properties and neuroprotective effects centrally and peripherally [30], and plays a vital role in IR regulation and glucose homeostasis in normal and neurodegenerative disorders like AD [62,63]. Lin et al. (2024) indicated that regular exercise training in high-fat diet and streptozotocin-induced Type 2 diabetes mellitus in rats effectively ameliorates IR and glucolipid metabolic dysfunction, and reduces inflammation in skeletal muscle by reductions in mitochondrial fission through the irisin/AMP-activated protein kinase signaling pathway [35].

It was reported that irisin reduced synaptic dysfunction and improved cognition by increasing the production of BDNF and other neuronal genes in AD, so that irisin can protect against neuronal damage by enhancing neurogenesis [62]. Furthermore, Sandoval and Gómez (2024) reported the anti-inflammatory effect of irisin in several studies, where this was associated with an upregulation of BDNF expression [64], while Huang et al. (2019) revealed that irisin could promote BDNF expression in an experiment on diabetic rats [65].

Importantly, irisin markedly reduced Aβ plaque as it increased the astrocytic release of neprilysin, the “Aβ-degrading enzyme” [38], with a subsequent abrogation of associated neuroinflammation and pathological circuits, along with an upregulation of the BDNF expression, leading to the hindrance of degenerative brain diseases [66,67].

Herein, the OVX/AD animals treated with MTN or/and PHE demonstrated a noteworthy upregulation in brain and serum irisin levels associated with an overall improvement in learning, memory, and biochemical and histopathological features of hippocampal biomarkers compared to the OVX/AD animals. They showed a notable increase in the percentage of SA, as well as an increase in the number of arm entries in the Y-maze test, which was associated with an increase in IGF-1 cell content and an upregulation of hippocampal PPAR-γ and BDNF protein expression, along with a marked decrease in hippocampal Aβ protein expression. Together, these inhibited brain neuroinflammation, manifested by the downregulation of TNF-α, NLRP3, and p38 MAPK content, leading to the abrogation of histopathological alterations and the neuroinflammation that suppresses microglial activation by repressing hippocampal GFAP content.

Collectively, this confirmed that irisin induction may have a critical role in AD treatment or prevention. Moreover, the significant decrease in the animals’ brain and serum irisin may explain the firing of neuroinflammatory signaling axes, as well as memory and cognitive deterioration observed in the current study, which paralleled the findings of previous studies [38,62]; whereas Kim et al. (2023) [38] used a three-dimensional cell culture model of AD to show that irisin markedly diminishes Aβ pathology by upregulating the astrocytic release of the Aβ-degrading enzyme neprilysin. Several investigations have shown that irisin induction has valuable therapeutic roles in neurodegenerative disease pathogenesis modulation, especially in AD [68,69].

Importantly, PPAR-γ is extensively distributed within multiple brain regions in astrocytes, microglia, and neurons [70]; in addition, the multifunctional nature of PPAR-γ allows it to modulate diverse cellular processes through its transcriptional activity. Moreover, PPAR-γ upregulation in neurodegenerative conditions stems from its ability to improve insulin sensitivity while simultaneously providing neuroprotective effects. Therefore, PPAR-γ modulation represents a promising avenue for preventing insulin-resistance-mediated AD [71].

Remarkably, irisin can act directly and indirectly on PPAR-γ, as it acts as an allosteric modulator or noncanonical ligand of PPAR-γ, by which underlying vital processes such as metabolic regulation, anti-inflammatory, and tissue-specific roles can be accomplished [72]. PPAR-γ can modulate astrocyte activation and morphology by directly downregulating GFAP expression through its gene promoter [73] or indirectly by downregulating the NLRP3 family, by modulating specific binding sites for PPAR-γ, located in the NLRP3 promoter regions [74,75]; Wang et al. (2015) [74] proposed that there is a strong correlation between PPAR-γ stimulation and inhibition of thiredoxin-interactive protein/NLRP3 inflammasome in a partial focal cerebral ischemic rat model. Collectively, the current results confirm the strong relationship between irisin, PPAR-γ activity, and neuroinflammation; therefore, PPAR-γ modulation by its agonists, i.e., irisin, may act as a promising target in neurodegenerative disorders, especially AD.

Importantly, Zhang et al. (2022) documented that irisin induced by PHE enhanced the memory function of elderly women with memory impairment via PPAR-γ co-activator-1α modulation [21]. Therefore, irisin is considered a bridge between PHE and neurological disorders. Moreover, in 2013, Wrann and collaborators confirmed that PHE improved fibronectin type III domain-containing protein 5 (FNDC5) expression in the hippocampus, from which irisin was cleaved, resulting in the elevated expression of BNDF in a way dependent on PPAR-γ co-activator-1α [18] and decreased Aβ protein formation [69,76]. Likewise, ref. [76] reported that physical and mental activities enhance brain neuroprotection against AD in rats by increasing BDNF, along with decreasing Aβ and pro-inflammatory markers such as TNF-α.

Notably, aerobic and swimming exercise upregulated PPAR-γ expression in muscle tissue, reduced muscle inflammation, and improved insulin-related pathways [77]. Aerobic exercise significantly upregulated IGF-1 expression in the gastrocnemius muscle of mice, as reported by [78].

Similarly, MTN is regarded as a new therapeutic avenue in AD due to its multiple mechanistic actions; it improves IR, controls energy balance, and enhances circulating irisin [30]. The decreased MTN receptor immunoreactivity within the hippocampi of AD patients was documented [79]. It was reported that MTN supplementation upregulated serum irisin levels in an OVX rat model [80]; another study [81] showed that MTN inhibited Aβ fibrillogenesis. In addition, Gáll et al. (2024) showed that MTN-treated animals exhibited lower brain content of TNF-α and GFAP than sham and diabetic animals [82]. Furthermore, it was stated that melatonin protects against N-methyl-D-aspartate-induced retinal ganglion cell injury by inhibiting the microglial TNFα-p38 MAPK pathway [83]. Additionally, MTN also promoted the expression of PPAR-γ and the signaling pathway of IGF-1 in rat pancreatic islets [84,85]. Moreover, MTN inhibited NLRP3 activation by a mitophagy-mediated reactive oxygen species [86]. Moreover, it upregulated BDNF expression in the cerebral cortex and hippocampus, which in turn improved cognitive impairment induced by sleep deprivation in rats [87].

## 4. Materials and Methods

### 4.1. Animals

Fifty female albino Wistar rats were used (12–16 weeks old, weighing 200 to 250 g) at the beginning of the experiment, obtained from the Faculty of Veterinary Medicine, Suez Canal University (SCU), Egypt. The animals were kept in stainless steel cages and experimental procedures were performed according to the Research Ethics Committee Faculty of Pharmacy, Cairo University (Approval No. PT3420), which follows the National Institutes of Health (NIH) Guide for the Care and Use of Laboratory Animals (Publication No. 8023, revised 2011).

### 4.2. Drugs and Chemicals

D-galactose (D-glc) and aluminum chloride (AlCl_3_) were purchased from (Sigma-Aldrich Chemical Co., St. Louis, MO, USA) and dissolved in 0.9% saline separately [88]. MTN was obtained from Puritan’s Pride Company (Holbrook, New York, NY, USA) and was dispersed in 0.9% saline with surfactant (1% Tween 80) [89]. All treatments were freshly prepared.

### 4.3. Study Design Compartments

#### 4.3.1. Induction of Ovariectomy

The rats were anesthetized by ketamine hydrochloride (100 mg/kg; i.p.) for the bilateral ovariectomy [90]. For sham animals, all ovariectomy procedures involved altering and exteriorizing the ovaries to induce a comparable degree of stress, but they were still kept, not removed [91]. The rats were housed in clean cages and observed daily for incision integrity, each receiving an oral suspension of amoxicillin (10 mg/kg, Amoun Pharmaceutical Co., El Obour City, Egypt) for 3 consecutive days after surgery [92], and an antibiotic cream was applied locally over the wound [90] to reduce postoperative infection.

#### 4.3.2. Induction of Alzheimer’s Disease

To induce AD, D-glc (60 mg/kg) and AlCl_3_ (10 mg/kg) were administered intraperitoneally daily for 42 days in the OVX rats [93].

#### 4.3.3. Swimming Physical Exercise Protocol

The rats were placed in a water tank (circular, 100 cm depth, 30 °C) maintained at 28–30 °C, and were permitted to swim for 5 days a week (from day 50 to 64); the duration of each session started with 5 min each day and then increased gradually to a maximum of a twenty minute “full session”. Every rat was forced to swim continuously and was prevented from floating [94]. The sham group animals spent the same time as the PHE groups in a shallow water tank that was 5 cm deep, with the absence of a workload to mimic the same level of stress as the PHE groups. Following each training session, they were towel-dried before being put back in their cages [95].

#### 4.3.4. Experimental Design

**Figure 7:**
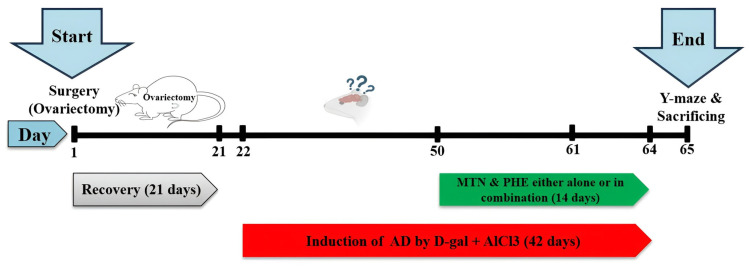
The schematic diagram illustrates the experimental design.

After animal recovery (3 weeks) from the surgical operation, the animals were aligned into 5 groups (ten rats for each group). The sample size was determined using G*Power version 3.1.9.7 software for a probable power calculation of a one-way ANOVA (fixed effects, omnibus test) using a significance level α (type-1 error) of 0.05, (1 − β-error probability) reflecting the power = 0.85, with the number of groups = 5, and effect size (f) = 0.55. Randomization was performed to allocate animals within groups using a computer-generated randomization list (https://www.graphpad.com/quickcalcs/randomize1/ (accessed on 1 February 2024)), where rats were assigned into 5 groups (10 animals/group). All groups except the sham group received daily intraperitoneal injections of D-glc (60 mg/kg) and AlCl_3_ (10 mg/kg) for 42 days to model AD in the OVX rats (OVX/AD). The MTN treatment and PHE sessions started in the last two weeks of the experiment and lasted until the end (from the 50th to the 64th day). The administration of all study drugs and PHE sessions was performed during the light phase between approximately 8:00 a.m. and 12:00 p.m. (Figure 7).

Group I (Sham): The rats were intraperitoneally administered 0.9% saline.

Group II (OVX/AD): The OVX animals received daily intraperitoneal injections of D-glc (60 mg/kg) then AlCl_3_ (10 mg/kg) for 42 days after the animal’s recovery and until the end of the experiment (from day 22 to 64) [93].

Group III (OVX/AD+PHE): The OVX/AD rats were exposed to swimming PHE for 14 days (from day 50 to 64) according to the PHE protocol described above.

Group IV (OVX/AD+MTN): The OVX/AD rats were administered MTN (10 mg/kg; i.p.) daily for 14 days (from day 50 to 64) [96].

Group V (OVX/AD+MTN&PHE): The OVX/AD animals were administered MTN and PHE by the same schedules as groups III and IV [95,96].

Start of the experiment: Bilateral surgical ovariectomy was achieved, followed by a 21-day recovery period.

Red arrow: D-galactose and aluminum chloride injection schedule for 42 days (from day 22 to 64).

Green arrow: Schedule of melatonin injection and physical exercise for 14 days (from day 50 to 64).

End of the experiment: Following an evaluation of the animals’ behavior using the Y-maze test, the animals were sacrificed on day 65.

### 4.4. Y-Maze Behavioral Test

On the last day of the experiment (65th day), the Y-maze test was performed in a Y-shaped apparatus (60 cm long and 10 cm wide) with three symmetrical arms, each labeled with letters (A, B, or C) in order to be differentiated. The rat was placed in one arm individually for 5 min to discover the maze freely, as well as the entry sequence of the arm. To get rid of any bias caused by odor, the maze was regularly cleaned with 70% ethanol between each rat. The spontaneous alternation (SA) score reflects spatial working memory and was assumed when the 3 arms, i.e., (BAC, CBA, and ABC), were successively entered. The formula for calculating the SA% is SA% = number of alternations/[(total no. of arm entries − 2)] × 100 [97]. Short-term spatial learning and memory function were directly proportional to the percentage of SA [98].

### 4.5. Tissue Sampling

On the last day (65 days) of the experiment, after evaluating the behavioral test for all animals, they were anesthetized with a single dose of thiopental (100 mg/kg; i.p.) [99], and eye puncture capillary tubes and serum separator tubes were utilized to obtain blood samples. The blood was centrifuged at 3000 rpm for 15 min and then allowed to stand for about 30 min. Following collection, the serum samples were preserved at −80 °C prior to biochemical analysis. Following the animals’ euthanization through cervical dislocation under anesthesia, rat brains were immediately extracted and washed with ice-cold isotonic saline. Brain tissue processing followed two distinct protocols: 4 whole brains/group were fixed in 10% neutral buffered formalin for 48 h to enable histopathological and immunohistochemical analysis of the hippocampal subiculum, while 6 hippocampi from the remaining brains were dissected and stored at −80 °C for subsequent biochemical analyses. To ensure unbiased results, a blinded investigator assessed the behavioral and histopathology/immunohistochemical analysis. Additionally, biochemical and histopathological samples were randomly selected.

### 4.6. Enzyme-Linked Immunosorbent (ELISA) Analysis

Biomarker quantification was performed using an enzyme-linked immunosorbent assay (ELISA) on 10% supernatants derived from the hippocampal subiculum tissues for the following: TNF-α (Cat No: SEA133Ra, Cloud-Clone Corp., Katy, TX, USA), p38 MAPK (Cat No: MBS720509), NLRP3 (Cat No: MBS7255410), GFAP (Cat No: MBS2505953), and IGF-1 (Cat No: MBS176012). The latter four biomarkers were obtained from (My BioSource, San Diego, CA, USA). However, irisin content was detected in both the serum and hippocampus supernatant (My BioSource, San Diego, CA, USA, Cat No: MBS2601445), and all ELISA procedures were conducted according to the manufacturers’ protocols and their established guidelines.

Protein content in the hippocampal supernatant was detected according to the recommendations of the Assay Kit of the Pierce™ BCA Protein Kit (Cat No: 23225, Thermo Fisher Scientific, Waltham, MA, USA).

### 4.7. Immunohistochemical Analysis

Four 4 μm thick formalin-fixed paraffin slices of hippocampi were prepared and incubated for immunostaining according to the procedures of [100,101] for the subsequent primary antibodies: Aβ, 1:100 (ABClonal, Woburn, MA, USA, Cat No: A17911); PPAR-γ, 1:100 dilution (ABClonal, Woburn, MA, USA, Cat No: A0270); and BDNF, 1:100 dilution (Servicebio, Wuhan, China, Cat No: GB11559). The primary antibodies were incubated overnight at 4 °C; following this, they were incubated and processed using a secondary antibody detection system for 60 min: the Mouse/Rabbit PolyDetector DAB HRP Brown Detection System (Bio SB, Santa Barbara, CA, USA, Cat No: BSB 0205). All hippocampal tissue sections underwent light counterstaining with hematoxylin for 30 s prior to the dehydration and mounting procedures. Microscopic examination was conducted at a magnification of 200×.

Both hippocampal neurons with nuclear reactions to BDNF and PPAR-γ and extracellular deposits with reactions to Aβ were considered positive. A semi-quantitative analysis of the stained tissue sections was accomplished by the modified Allred scoring system guidelines [102]. The number of positive neurons and the percentage of extracellular deposits in three high-power fields (400×) were counted using ImageJ software 1.54p (ImageJ, NIH, Bethesda, MD, USA, RRID: SCR-0030) [103], and the mean +/− standard deviation were calculated. Individual scores for the percentage of positive cells (0–5) were summed to obtain the final grades, and the percentage of positive cells was established as follows: 1—less than 10; 2—from 10 to 20; 3—from 20 to 50; 4—from 50 to 70; and 5—more than 70.

### 4.8. Histopathological Examination of Brain Tissue Using Hematoxylin and Eosin Stain

Paraffin-embedded tissue sections of hippocampal specimens were cut to 3 μm thickness and subjected to hematoxylin and eosin (H&E) staining according to the procedures of [100,101]. Different hippocampi sections for each group were observed blindly by two qualified pathologists at 200× to assess histopathological alterations, i.e., neuronal pyknosis, peri-neuronal edema, areas of necrosis, chronic inflammatory cell infiltrates, and reactive gliosis. The severity of microscopic lesions detected in the hippocampus was graded according to the degree and extent of tissue damage using a 4-point scale, as previously described: (absent (grade 0)—no lesions detected; minimal (grade 1)—lesions involved less than 15% of the tissue section; mild (grade 2)—lesions involved 15–45% of the tissue section; moderate (grade 3)—lesions involved 45–75% of the tissue section; marked (grade 4)—lesions involved greater than 75% of the tissue section) [104]. Then, the data are presented as median (Min to Max) ± SD. [100].

### 4.9. Statistical Analysis

The normality of data distribution was assessed using the Shapiro–Wilk test. The data are presented as the mean ± standard deviation, with statistical significance set at *p* < 0.05. For the comparison of multiple groups, one-way analysis of variance (ANOVA) was employed, followed by the Tukey–Kramer post hoc test for multiple comparisons. However, histopathological evaluations were analyzed by a non-parametric Kruskal–Wallis test, with subsequent Mann–Whitney U tests, and the data are presented as median (Min to Max) ± SD. All statistical computations were performed using GraphPad Prism software (version 9, GraphPad Software, Inc., San Diego, CA, USA), and the effect sizes (η^2^ = SS effect/SS total) for the ANOVA were calculated (η^2^: large = 0.14, medium = 0.06, small = 0.01). Correlation analyses were conducted using Pearson’s correlation coefficient with a 95% confidence interval, considering correlation significance at *p* < 0.0001. To control for type I error resulting from multiple comparisons, *p*-values were adjusted using the Benjamini–Hochberg false discovery rate (FDR) method in GraphPad Prism. Statistical significance was defined as adjusted *p*-values (q-values) < 0.05 [105].

## 5. Conclusions

The current study showed that irisin induction by MTN or/and PHE may open new therapeutic avenues in neurodegenerative disorders, especially AD, highlighting the strong correlation between IR and neuroinflammation in the pathogenesis of AD incidence and progression in menopausal women. Thus, irisin may attenuate AD progression by upregulating PPAR-γ and IGF-1 in addition to inhibiting brain neuroinflammation (TNF-α/p38 MAPK/NLRP3/GFAP), which results in enhancing BDNF and suppressing Aβ expression in the hippocampus.

### Study Recommendations

Further studies are encouraged to study the potential effects of the current treatment in various rodent models of AD, using diverse animal strains, sexes, and ages, either using the current MTN schedule or a different regimen to ensure study safety and efficacy in the long term. Moreover, we encourage employing irisin/FNDC5 inhibitors, siRNA knockdown, and molecular validation (protein/gene expression).

## Data Availability

The original contributions presented in this study are included in the article. Further inquiries can be directed to the corresponding author.

## Abbreviations

The following abbreviations are used in this manuscript:

Aβ	Amyloid beta
AD	Alzheimer’s disease
AlCl_3_	Aluminum chloride
BDNF	Brain-derived neurotrophic factor
D-glc	D-galactose
FNDC5	Fibronectin type III domain containing 5
GFAP	Glial fibrillary acidic protein
IGF-1	Insulin-like growth factor 1
IR	Insulin resistance
IL	Interleukin
MTN	Melatonin
NLRP3	NOD-like receptor protein 3
OVX	Ovariectomized
PPAR-γ	Peroxisome proliferator-activated receptor gamma
p38 MAPK	p38 mitogen-activated protein kinase
PHE	Physical exercise
SA	Spontaneous alternation
TNF-α	Tumor necrosis factor-alpha
